# Rapoport's Rule Revisited: Geographical Distributions of Human Languages

**DOI:** 10.1371/journal.pone.0107623

**Published:** 2014-09-12

**Authors:** Michael C. Gavin, John Richard Stepp

**Affiliations:** 1 Department of Human Dimensions of Natural Resources, Colorado State University, Fort Collins, CO, United States of America; 2 Department of Anthropology, University of Florida, Turlington Hall, Gainesville, FL, United States of America; University of Cambridge, United Kingdom

## Abstract

One of the most well studied ecological patterns is Rapoport's rule, which posits that the geographical extent of species ranges increases at higher latitudes. However, studies to date have been limited in their geographic scope and results have been equivocal. In turn, much debate exists over potential links between Rapoport's rule and latitudinal patterns in species richness. Humans collectively speak nearly 7000 different languages, which are spread unevenly across the globe, with loci in the tropics. Causes of this skewed distribution have received only limited study. We analyze the extent of Rapoport's rule in human languages at a global scale and within each region of the globe separately. We test the relationship between Rapoport's rule and the richness of languages spoken in different regions. We also explore the frequency distribution of language-range sizes. The language-range area distribution is strongly right-skewed, with 87% of languages having range areas less than 10,000 km^2^, and only nine languages with range areas over 1,000,000 km^2^. At a global scale, language-range extents and areas are positively correlated with latitude. At a global scale and in five of the six regions examined, language-range extent and language-range area are strongly correlated with language richness. Our results point to group boundary formation as a critical mediator of the relationship between Rapoport's rule and diversity patterns. Where strong group boundaries limit range overlap, as is the case with human languages, and range sizes increase with latitude, latitudinal richness gradients may result.

## Introduction

Spatial patterns in the range sizes of species have been a major focus of research in macroecology and biogeography because of the potential implications for conservation efforts and for the origin and maintenance of biodiversity patterns [1]–[6]. One of the most well studied patterns is Rapoport's rule, which posits that the geographical extent of species ranges increases at higher latitudes. In the twenty years since the first introduction of Rapoport's rule [6], over 100 studies have investigated this biogeographical pattern [7]. Smaller range sizes at lower latitudes may help explain the propensity of most taxa to demonstrate greater diversity in the tropics; and explaining this diversity gradient is the ‘holy grail’ of biogeography [8]. Stevens [6] noted that Rapoport's rule may be a critical underlining mechanism explaining latitudinal gradients in species diversity.

However, despite all the research on Rapoport's rule, nearly all the studies to date have been limited in their geographic scope [7], [9]. Results of these empirical tests of Rapoport's rule have been equivocal. Rohde [10] concluded that the effect is only a local phenomenon, and others have noted that the effect is mostly restricted to northern latitudes in North America [3], [11]. Ruggiero and Werenkraut [7] conducted a meta-analysis of 49 studies and found that Rapoport's rule varies greatly across different regions. Therefore, more global scale analyses are required that can capture the spatial variation in Rapoport's rule for major taxa and, in turn, examine the relationship between geographic range sizes and macroscale patterns in diversity [9].

**Figure 1:**
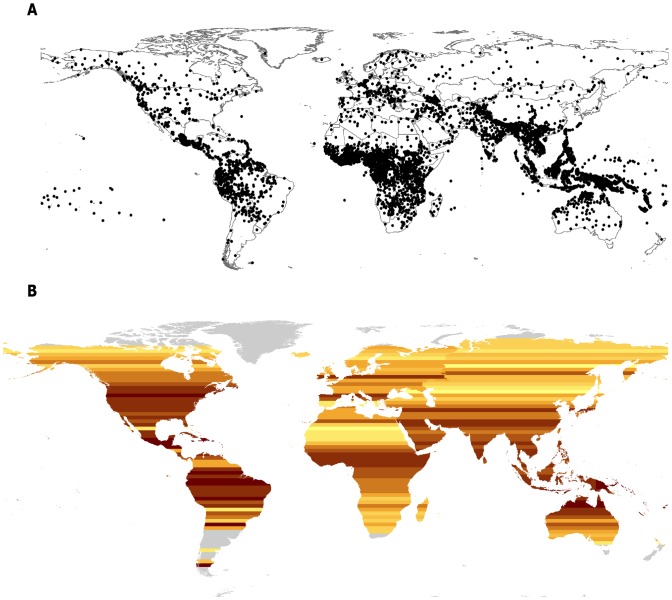
Distribution of the world's languages. Each point represents the center of the range of one language.

Humans collectively speak almost 7000 different languages [12], [13]. This language diversity is spread unevenly across the globe, with loci in the tropics (Fig. 1, 3C), particularly in Mesoamerica, Equatorial Africa, Southeast Asia, and the Pacific. Many mechanisms for human socio-linguistic and cultural diversification have been proposed focusing on historical, ecological, economic, political, and social drivers [14]–[28]. Only a limited number of studies have empirically tested the proposed mechanisms [28], [29], and the results to date have been equivocal, pointing to different possible mechanisms depending on the scale of the study (e.g., global vs. regional), the region of focus (e.g., South America vs. the Pacific), and the analytical methods employed (e.g., simple correlation, multiple regression, regression trees). The search for an explanation for geographic patterns in language diversity can benefit from hundreds of previous studies in biogeography, which have studied similar patterns in species richness and draw from a rich history of empirical analysis.

The existence (or not) of Rapoport's rule in relation to human socio-linguistic diversity has received surprisingly little attention. Mace and Pagel [30] provide the only direct measure of Rapoport's rule for human languages with evidence for an increase in latitudinal range extents at higher latitudes in North America, which corroborates findings for other North American mammals [6], [31], [32]. Both Nettle [33] and Currie and Mace [16] note a similar trend in other regions (West Africa and the Old World respectively), but do not test the effect size. Likewise, no research has explored empirically the relationship between the geographic patterns in language-range sizes and the obvious latitudinal gradients in language diversity. Currie and Mace [16] note a possible link between average range size and diversity, but they do not test the relationship statistically. Our study is the first to conduct a global analysis at multiple scales of the spatial variation in human language-range extent and area sizes. We analyze the magnitude of Rapoport's rule in human languages at a global scale and within each region of the globe separately. We also test the relationship between Rapoport's rule and the diversity of human languages spoken in different regions. In addition, we explore the frequency distribution of language-range sizes and examine the significance of this distribution for our understanding of human diversity. Our study serves to both test the applicability of Rapoport's rule across multiple scales, and to examine the degree to which Rapoport's rule can help explain the latitudinal gradient in language diversity.

## Results

### Language-Range Area Distribution

**Figure 2:**
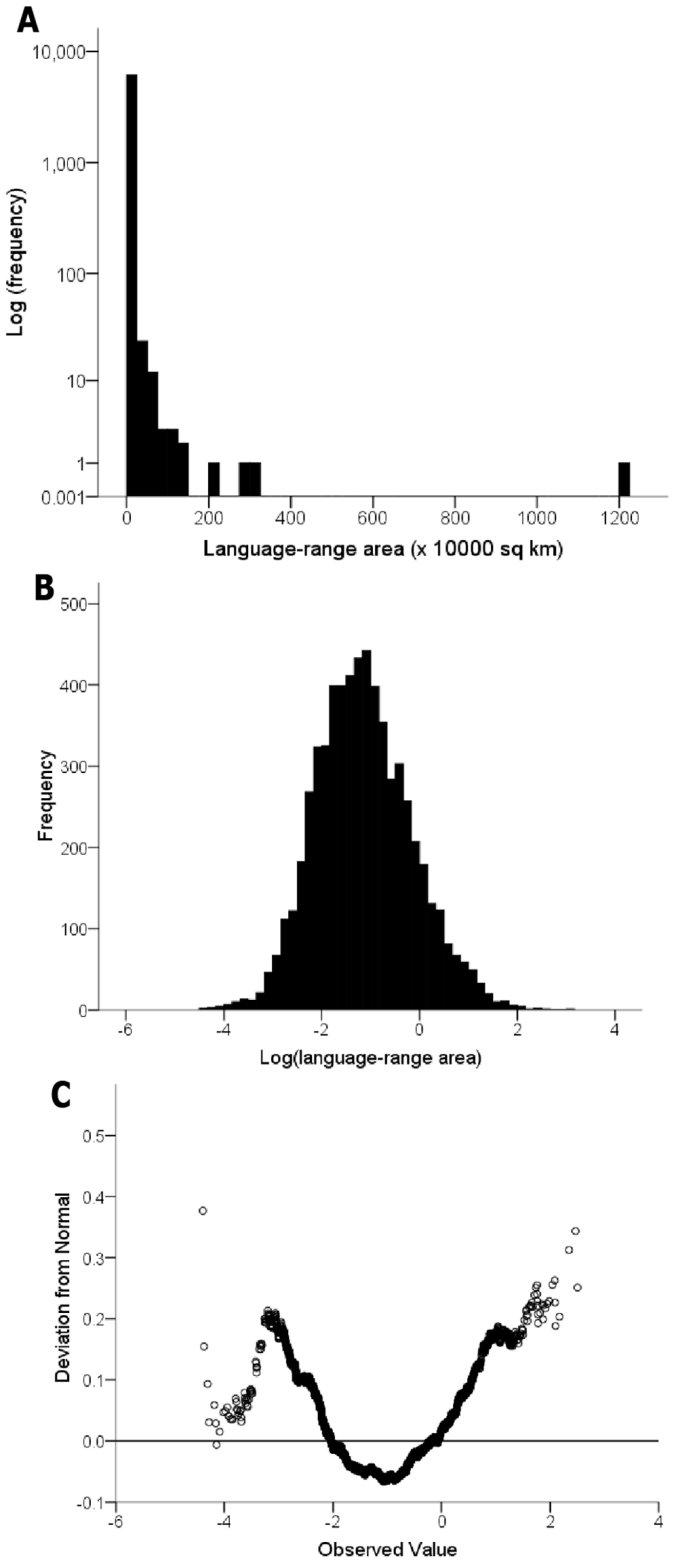
Language-range area distribution. (A) Untransformed range areas (note: y-axis is log(frequency)). (B) Log-10 transformed range areas. (C) Deviation from normal following log-10 transformation.

The language-range area distribution is strongly right-skewed (Fig. 2). 87% of languages have a range area less than 10,000 km^2^, while only nine languages have a range area over 1,000,000 km^2^. The language-range area distribution is not formally log-normal (D(7219) = .025, P<.001) with the range areas of both the most range-restricted languages and the most widespread languages larger than expected (Fig. 2C).

### Phylogenetic Autocorrelation

Variance components analysis demonstrates that the majority of the variation in language-range extent is found at the level of individual languages (84.8%), with only 15.2% at the level of language families. For language-range area 99% of the variation is at the level of individual languages, with only 1% at the level of language families. Therefore, we conclude that language-range sizes show relatively weak phylogenetic dependence, and we report all results based on analyses at the level of individual languages.

### Latitudinal Variation in Language-range Sizes

**Figure 3:**
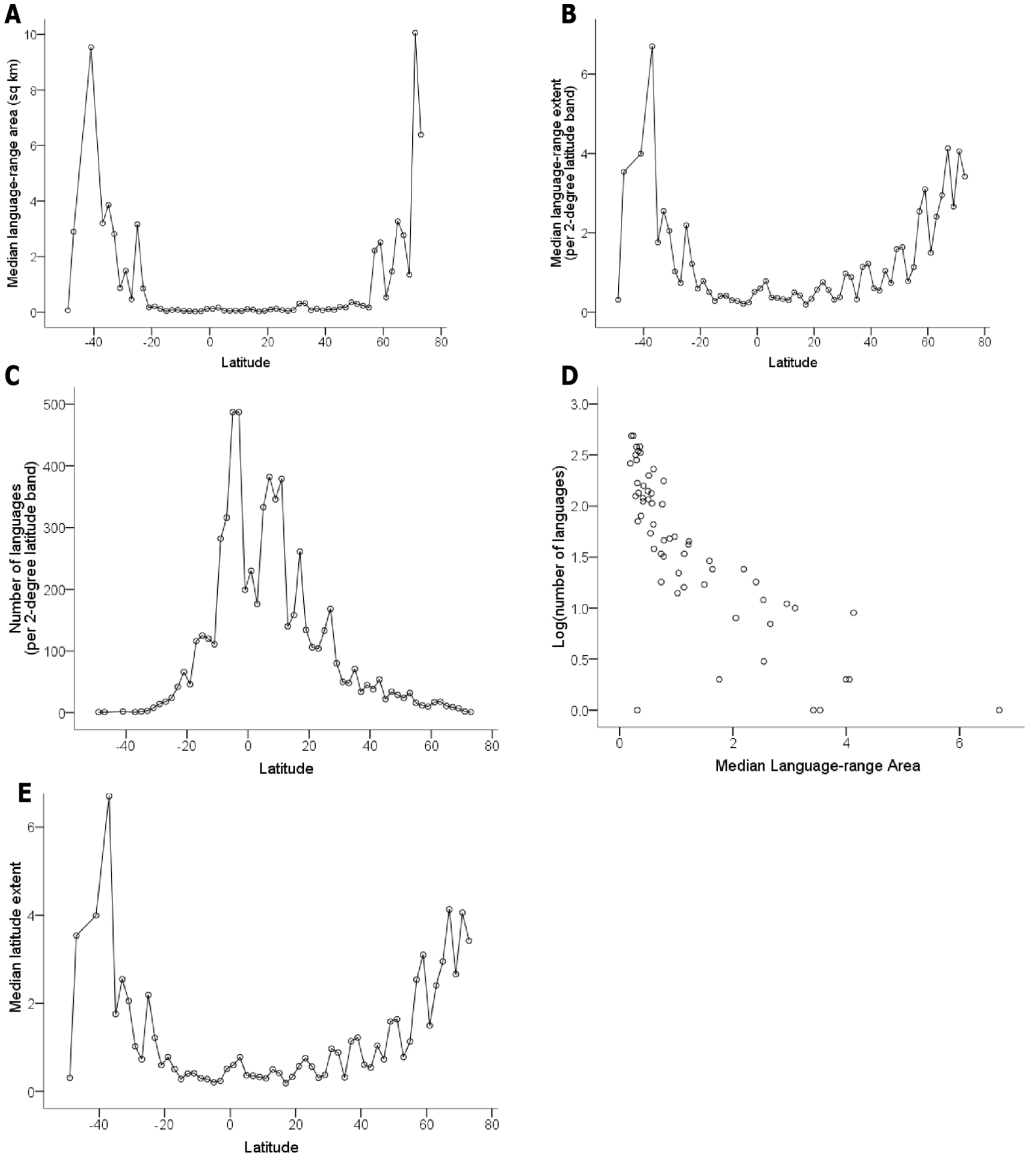
Global relationships between language range area, language range extent, language richness, and latitude. (A) Changes in median language-range area (determined with mid-point method with 2 degree bands) at different latitudes (Correlation coefficient (using absolute latitude and Spearman's rank correlation): 0.63) (B) Changes in median range extent (determined with mid-point method and 2-degree lat bands) of language at different latitudes (Correlation coefficient (using absolute latitude and Spearman's rank correlation): 0.69) (C) Latitudinal gradient in language richness (number of languages per 2-degree latitude band) (D) Median language-range area versus log-language richness.

At a global scale language-range extents and areas are strongly positively correlated with latitude (Table 1 and Fig. 1B, 3A, 3B). The relationships between latitude and language-range extent and area are moderate to strong in all regions except Europe and South America. The limited number of latitudinal bands in Europe may reduce the statistical power of the midpoint method. Examining each indigenous language as an independent point [34], both language-range extent (Europe: n = 151 languages, r_s_ = 0.20, P<.05; South America: n = 395 languages, r_s_ = 0.14, P<.01) and language-range area (Europe: r_s_ = 0.25, P<.01; South America: r_s_ = 0.1, P<.1) are correlated with latitude in Europe and South America. The correlation coefficients are smaller with the independent points method, which is to be expected given the challenges the method faces with the introduction of dispersion into the data [7]. All results were also similar using Stevens [6] original method (e.g., global scale correlation between language-range extent and latitude, r_s_ = 0.9, P<.01).

**Table 1:** Spearman rank correlations between language-range sizes and latitude and language richness.

Region	Range Latitude Extent and Absolute Latitude	Range Area and Absolute Latitude	Range Latitude Extent and Language Richness	Range Area and Language Richness	Sample size (total latitudinal bands included)
Global:					
1-degree	**0.75**	**0.44**	**−0.81**	**−0.70**	111
2-degree	**0.73**	**0.67**	**−0.85**	**−0.85**	62
5-degree	**0.79**	**0.74**	**−0.94**	**−0.96**	25
N. Hemisphere:					
1-degree	**0.79**	**0.74**	**−0.86**	**−0.83**	73
2-degree	**0.83**	**0.80**	**−0.90**	**−0.88**	37
5-degree	**0.91**	**0.85**	**−0.94**	**−0.89**	15
S. Hemisphere:					
1-degree	**0.79**	**0.74**	**−0.86**	**−0.83**	73
2-degree	**0.76**	**0.77**	**−0.82**	**−0.82**	25
5-degree	**0.90**	**0.95**	**−0.95**	**−0.95**	10
Africa:					
1-degree	**0.62**	**0.68**	**−0.84**	**−0.87**	64
2-degree	**0.57**	**0.66**	**−0.80**	**−0.87**	35
5-degree	**0.55**	**0.75**	**−0.79**	**−0.93**	15
Asia:					
1-degree	**0.71**	**0.76**	**−0.70**	**−0.73**	79
2-degree	**0.79**	**0.81**	**−0.75**	**−0.76**	43
5-degree	**0.88**	**0.88**	**−0.67**	**−0.66**	18
Europe:					
1-degree	ns	ns	−0.40	−0.42	36
2-degree	ns	ns	−0.48	ns	18
5-degree	ns	ns	−0.79	ns	7
Pacific:					
1-degree	**0.89**	**0.89**	ns	ns	43
2-degree	**0.90**	**0.92**	ns	ns	25
5-degree	**0.91**	**0.91**	ns	ns	12
N. America:					
1-degree	**0.39**	**0.34**	**−0.62**	−**0.51**	52
2-degree	**0.62**	**0.48**	**−0.77**	**−0.75**	29
5-degree	**0.80**	ns	**−0.77**	**−0.68**	22
S. America:					
1-degree	ns	**0.37**	−.30	−0.31	44
2-degree	ns	ns	**−0.5**	ns	24
5-degree	ns	**0.79**	**−0.78**	**−0.88**	12

All correlations in boldface type significant at P<.01; otherwise significant at P<.05; ns, nonsignificant.

### Language-range Sizes and Language Richness

Language richness is strongly correlated with latitude, with far more languages in the tropics than at higher latitudes (Fig. 1A, 3C). At a global scale, as well as in each hemisphere and in five of the six regions examined (the exception being the Pacific), language-range extent is negatively correlated with language richness (Table 1, Fig. 3D, 3E). Language range area is also negatively correlated with language richness in every region except Europe and the Pacific.

## Discussion

The vast majority of languages are spoken in very limited areas and, as with many other taxa [35], the frequency distribution of language-range sizes approximates log-normal. However, the most widespread and the most geographically restricted languages have range sizes larger than expected (Fig. 2C). The absence of languages with very small range sizes may be due to under-sampling of these groups or because of proportionately higher rates of extinction. Very small ranges likely lead to greater exposure to other languages and higher probability of language shifts. In addition, smaller areas will have relatively fewer resources and, in turn, may support only limited populations. If group size falls too low, ethnolinguistic groups may suffer from a version of the Allee effect (i.e. decreased individual fitness with lower population size or density), which can contribute to the higher extinction rates of smaller populations [36]. The most widely dispersed languages may owe their large range size to the extraordinary migratory ability of humans. Through cultural and technological innovation (agriculture, maritime navigation, etc), our species has been able to spread rapidly and survive in nearly every biome on the planet and to adapt to a broad array of different ecological conditions.

Unlike studies of other taxa [7], [10], we found evidence for Rapoport's rule at a global scale and in most regions of the globe. We also found support for Stevens' [6] original assertion that Rapoport's rule relates to latitudinal diversity patterns. But why would Rapoport's rule be more pronounced in humans than in many other taxa; and what mechanisms might be responsible for the relationship between Rapoport's rule and the latitudinal gradient in language diversity?

Stevens [6] proposed that the link between Rapoport's rule and the latitudinal gradient in species richness was due to Rapoport's rescue effect. Drawing on Jansen's [37] previous hypothesis related to tropical mountain passes, Stevens [6] proposed that species evolving in the tropics had lower climatic tolerances (i.e. range of temperature and precipitation), which would limit their latitudinal ranges. In addition, Stevens argued that demographic sink areas would exist outside the normal range of species; and these areas would only support individuals of the species due to immigration from the range core. He argued that the overlap of demographic sink areas caused the increase in species richness in tropical regions. Stevens' hypothesized mechanism has led to ample debate within biogeography [38]. Gaston and Chown [39] pointed out a critical limitation by noting that key climatic variables, such as temperature, tend to level off across the tropics, while species richness in the region still varies considerably with latitude. Taylor and Gaines [40] simulated species ranges and found that Rapoport's rule alone or in combination with demographic sink perimeters did not produce the expected latitudinal gradient in species richness. In fact, in some cases the models produced higher richness at the poles. However, when Taylor and Gaines [40] included a measure of competition in their model the result matched the observed greater species richness in the tropics. Because competition limited the overlap of species ranges in the model, the authors attributed the geographic patterns produced to ‘species packing’, in which more species of smaller ranges can be packed into a similar area.

Our results provide empirical evidence in support of the theoretical models of Taylor and Gaines [40] and have clear implications for understanding latitudinal patterns in language diversity. Unlike most species ranges for the taxa considered to date, human language ranges do not tend to overlap. For example, whereas a given location on the globe may support several different mammal species, each location tends to support only one human language. In the database used in this study, two or more languages co-exist on only 5% of the land area that support humans. As was the case in the simulations of Taylor and Gaines [40], this constraint on language range overlap helps explain the strong correlation between language range size and language richness. Therefore, we suggest that two key factors lead to the latitudinal gradient in language diversity: Rapoport's rule and the strong group boundary formation between socio-linguistic groups that prevents overlap. Understanding the mechanisms underlying these two processes is the next critical step to explaining the drivers of geographic patterns in language diversity.

### What leads to Rapoport's rule with languages?

Gaston et al. [11] review five mechanisms proposed as causes for Rapoport's rule with other species: land area, differential extinction, competition, climatic variability, and geographic boundaries. Land area does not seem to be a likely contributor in the case of language ranges. Although land area is greater at higher latitudes in some regions (e.g., Pacific; r_s_ = 0.73, p<.001), the trend is the opposite in other regions (e.g., Africa; r_s_ = −0.44, p<.01) where we still record a significant Rapoport's effect for languages.

Variable rates of extinction are also an unlikely mechanism for the formation of Rapoport's effect with languages. The impact of many of the extinction vectors for languages (e.g., climate change, glaciation, European colonial expansion) vary enormously in different regions of the globe, but Rapoport's effect occurs to a similar extent in most regions. For example, colonial expansion had a massive impact on indigenous New World populations as compared to parts of the Old World, but Rapoport's effect is evident in both regions. Alternatively, competition may play a critical role, and the causal relationship between range size and diversity may be in the reverse direction with greater diversity at lower latitudes leading to greater competition, which then leads to smaller ranges. However, if this is the case, then the question of where language richness originates from would still remain.

Stevens [6] proposed that seasonal climates at higher latitudes favour individuals with greater tolerance for environmental variation, and therefore species at these latitudes have larger ranges. Although humans have only a limited amount of physiological plasticity, different technological advances (e.g., specialised clothing, diverse resource use, domestication of seasonal grain crops) may have allowed some groups to better cope with seasonal conditions, and therefore to expand their ranges in regions with variable climates. Although this proposed mechanism of technological innovation may explain the shift to substantially larger ranges at higher latitudes, we still detect a clear Rapoport's effect when we limit our analysis to latitudinal bands between 20N and 20 S (language-range extent and absolute latitude, r_s_ = 0.63, p<.01; language-range area and absolute latitude, r_s_ = 0.68, p<.01). Gaston and Chown [39] also noted this same issue with Steven's argument, pointing out that key environmental variables (e.g., mean annual temperature) do not vary considerably within the tropics but in this same area species ranges tend to vary with latitude. This evidence implies that more investigation is needed into the role of specific technological advances on geographic patterns of language-range sizes, but that this mechanism alone may not be responsible for the entire global pattern in range sizes we record here.

Biogeographical boundaries may also constrain language ranges. If human subsistence strategies are linked to particular biogeographic zones (e.g., it is difficult to farm in the tundra), then the size of language ranges may be limited by biogeographic boundaries. In turn, because biogeographical units are larger at higher latitudes [11], language-range sizes would correspond with Rapoport's effect. Although no direct tests of this hypothesis have been performed for human languages, Currie and Mace [16] show that different subsistence strategies are practiced at significantly different latitudes. On a related note, a greater density of biogeographic units at lower latitudes would also create more biogeographic edges, and Turner et al [41] suggest that cultural groups may actively use these edges to maximise resource availability. Therefore, of the major mechanisms Gaston et al. [11] outlined for Rapoport's effect, the mechanisms related to climatic variability and geographic boundaries appear to have the greatest potential to provide a non-circular argument that can explain at least some of the global patterns in language-range sizes and language diversity.

Ultimately, language diversification may be a social process, in which speakers make a choice either to shift their language or to maintain their current means of communication. This choice can be impacted by a plethora of social factors, including discrimination, repression, war, immigration, religion, compulsory education, and stigmatization [42]. However, the strong latitudinal gradient in language-range sizes and language diversity points to diversification mechanisms that also have some environmental component. This does not rule out social factors playing a critical role. Environmental conditions may ‘set the stage’ for social processes that cause diversification or convergence of languages [43].

For example, Nettle [20] argues that language diversity patterns are shaped by the combined effect of social networks and ecological risk. He notes that linguistic norms are spread through social networks. Nettle posits that the size of social networks and the area they cover relates to ecological risk. He argues that more stable environments with longer growing seasons provide a more stable resource base. In turn, language groups in aseasonal climates may require smaller social networks, relying less on trade with neighbouring groups because of abundant local resources, and also use less land area. Therefore, less climatic variation at lower latitudes would support smaller social networks, smaller language ranges and greater language diversity. In seeking empirical support for his hypothesis Nettle restricted his analysis to non-tropical regions, and subsequent tests of the hypothesis [16], [36] have found little evidence for a strong link between mean growing season and either language diversity or language-range size. Part of the challenge may be in the definition of ecological risk, given that growing season only captures variation in precipitation and temperature, but numerous other factors (soil fertility, habitat heterogeneity, etc) combine to determine the quantity and variety of resources that can be grown or gathered in a given location.

Another example of the interplay between environmental and social drivers of language diversification and language-range sizes involves the development of grain-based agriculture in different temperature regions of the world that required the presence of particular environmental conditions and potential cultivars. In some regions, agriculture supported the formation of larger and more politically “complex” societies, in which cultural traits, such as language, tended to homogenise [16], [17]. Through more intensive resource use and more coordinated actions of a larger number of people, these “complex” societies could maintain a competitive advantage eventually absorbing or extinguishing their less politically complex neighbours [16], [17]. Currie and Mace [16] found that political complexity positively correlates with language-range size, that political complexity varies on a latitudinal gradient, and that variance in political complexity can explain a portion of the spatial variation in language diversity. The role of political complexity also demonstrates the intricacy of the mechanisms likely involved in determining language-range sizes and language diversity patterns, as environmental variables (including the placement of biogeographic boundaries) limited agricultural development, which in turn led to political complexity and subsequent cultural changes at a landscape scale. Likewise, the development of agriculture and political complexity did not follow the same temporal or spatial trajectory in each hemisphere or on each continent; and therefore it is not surprising that this variable alone explains less than half the spatial variation in language diversity.

### What leads to group boundary formation?

Boundaries between human groups can be created by both biophysical (e.g. oceans, mountains) and social (e.g. castes, clans) barriers. In a recent paper [29], along with other colleagues, we outlined in detail the major factors (movement, contact, selection, and neutral change) that contribute to the formation or deterioration of group boundaries. For example, movement of human groups into new, isolated environments (e.g. oceanic islands or remote mountain valleys) can create separation from other groups. If this separation occurs over enough time, neutral changes in heritable language units can accumulate and lead to language diversification. In addition, movement can lead to contact between groups of people. Many different outcomes can result for the ranges of the languages spoken by the people who come into contact, including no changes in language ranges, one language or group of speakers displacing another, linguistic change via borrowing, or the formation of new languages (e.g. creoles). Also, social and environmental conditions can contribute to variability in social status within populations, which can create group boundaries and differences in language use. What remains unknown, and an area in need of future research focus, is the degree to which these different processes shape group boundaries across space and time.

## Conclusion

Overall, our results have two important implications. First, we contribute to the long-standing debate regarding the role of Rapoport's rule in shaping latitudinal patterns in diversity. In support of theoretical models of Taylor and Gaines [40], we provide empirical evidence that Rapoport's rule may help explain latitudinal diversity patterns when combined with strong constraints on range overlap. These constraints are clearly apparent with the ranges of human languages, which overlap to only a very limited extent (<5% of total area), but not as apparent with other taxonomic groups, which may explain the lack of a strong link between Rapoport's rule and diversity patterns with certain taxa in particular regions.

In addition, our results contribute to the small but growing body of literature examining drivers of geographic patterns in language diversity. We conclude that future research on geographic patterns of human diversity would benefit from more in-depth analysis of the drivers of language-range sizes and group boundary formation, and the mechanisms which link these variables to language diversity. To date, only one study [16], has conducted an empirical analysis of possible drivers of language range sizes, and this covered less than 10% of the world's languages. We also note that not one single “silver bullet” factor exists to explain all the spatial variation in language-range sizes and language diversity. To understand the complex web of variables involved in determining geographic patterns in language diversity, we argue that future research must examine the role of multiple predictors (and their interactions) operating at different spatial and temporal scales. Recent advances in simulation modelling [16], [44], [45] can also explicitly incorporate diversification mechanisms into predictive models. Finally, any study of patterns in human cultural diversity must also recognise the rapid pace of cultural change affecting the world's languages (i.e. a rate of language loss many times faster than the rate of biodiversity loss, [36]). By some estimates, 90% of the world's languages will be gone in less than a century [46], a fact that must be a call to action to avoid the loss of countless years of cultural heritage in just one lifespan.

## Methods

### Data

We used digital language maps from Global Mapping International (http://www.gmi.org) to examine language ranges. The maps contain data from the fifteenth edition of the Ethnologue [12], which represents the most comprehensive catalogue of the languages spoken in the world. We calculated language-range areas using ArcGIS [47] and defined the latitudinal extent of languages as the latitudinal span between the maximum and minimum latitude in the range (i.e., a language ranging from 10S to 10N latitude would have a latitudinal extent of 20).

### Analysis

Ruggiero and Werenkraut [7] demonstrate that the method used to study Rapoport's rule can have a significant impact on results. We used Rhode's [48] midpoint method to examine Rapoport's rule because the method avoids issues with spatial non-independence inherent in Steven's [6] original method. We calculated the median language-range extent and language-range area for languages with midpoints falling within each 1-degree latitudinal band (e.g., 0–1N), and repeated the analysis for 2-degree and 5-degree latitudinal bands. We also tallied the number of languages with midpoints in each band as a measure of language richness. Because the distribution of language-range extents, language-range areas, as well as language richness, were non-normal (analysed using Kolmogorov-Smirnov test, see results and Fig. 1), we used Spearman's correlation coefficients to analyse the relationship between latitude and range extent, latitude and range area, latitude and language richness, range extent and language richness, and range area and language richness. We ran correlations first at a global scale and then in each major region (see Table 1). The spatial patterns of language-range sizes may be confounded by phylogeny. Because resolved language phylogenetic trees are unavailable for many language families, we did not employ phylogenetic comparative analysis. Instead, we tested for phylogenetic dependence using variance components analysis [49] to compare the amount of variation in language range sizes evident at the level of language families (data from Ethnologue [12], and among individual languages.
